# Supplementary material for: The Human Myelin Proteome and Sub-Metalloproteome Interaction Map: Relevance to Myelin-Related Neurological Diseases
Source: Brain Sci. 2022 Mar 24;12(4):434. doi: 10.3390/brainsci12040434 (PMC9029312; doi:10.3390/brainsci12040434)
Supplement: Supplementary file 1 [file brainsci-12-00434-s001.zip › Contents of Supplementary material.pdf]

Contents of Supplementary Material

The Human Myelin Proteome and Sub-Metalloproteome Interaction Map: Relevance to Myelin-Related Neurological Diseases

Christos T. Chasapis [1,2,*], Konstantinos Kelaidonis [3], Harry Ridgway [4,5], Vasso Apostolopoulos [6,7] and John M. Matsoukas [3,6,8,*]

[1] NMR Facility, Instrumental Analysis Laboratory, School of Natural Sciences, University of Patras, 26504 Patras, Greece
[2] Institute of Chemical Engineering Sciences, Foundation for Research and Technology, Hellas (FORTH/ICE-HT), 26504 Patras, Greece
[3] NewDrug PC, Patras Science Park, 26504 Patras, Greece; k.kelaidonis@gmail.com
[4] Institute for Sustainable Industries and Liveable Cities, Victoria University, Melbourne, VIC 3030, Australia; ridgway@vtc.net
[5] AquaMem Scientific Consultants, Rodeo, NM 88056, USA
[6] Institute for Health and Sport, Victoria University, Melbourne, VIC 3030, Australia; vasso.apostolopoulos@vu.edu.au
[7] Immunology Program, Australian Institute for Musculoskeletal Science (AIMSS), Melbourne, VIC, Australia
[8] Department of Physiology and Pharmacology, Cumming School of Medicine, University of Calgary, Calgary, AB T2N 4N1, Canada
* Correspondence: cchasapis@upatras.gr (C.T.C.); imats1953@gmail.com (J.M.M.)

1. **Table S1_PPI_network**

sheet "Network": Binary interactions of the myelin proteins with UniProt IDs

sheet "Degree": Degree of connectivity for all myelin proteins involved in PPI network

sheet "Top 10": The top 10 proteins in myelin PPI network that have the largest number of interactions (>24)

2. **Table S2_Proteomes**

sheet "MYELIN_PROTEOME": UniProt IDs, protein names, protein function, gene names of myelin proteins

sheet "MYELIN_METALO_PROTEOME": Total metal binding proteins involved in PPI network

sheet "Calicium_proteome": Calcium binding proteins

sheet "Cobalt_proteome": Cobalt binding proteins

sheet "Copper_proteome": Copper binding proteins

sheet "Iron_proteome": Iron binding proteins

sheet "Magnesium_proteome": Magnesium binding proteins

sheet "Manganesse_proteome": Manganesse binding proteins

sheet "2Fe-2S_proteome": 2Fe-2S binding proteins

sheet "3Fe-4S_proteome": 3Fe-4S binding proteins

sheet "4Fe-4S_proteome": 4Fe-4S binding proteins

sheet "Iron_sulfur_proteome": Iron_sulfur binding proteins

sheet "Zinc_proteome": Zinc binding proteins
